# Anomalies of Refraction—A Case of Cross-eyes

**Published:** 1886-12

**Authors:** R. O. Cotter

**Affiliations:** Macon, Ga.


### ANOMALIES OF REFRACTION—A CASE OF CROSS-EYES.

BY R. O. COTTER, M. D., MACON, GA.

The subject of squint or cross-eyes, its causes, prevention and treatment, though belonging more appropriately in a journal devoted to ophthalmology, is a most important one to the general practitioner also. Convergent squint (eyes crossed inward) is largely caused by hypermetropia; practically the antero-posterior diameter of the eyeball is too short. Parallel rays cannot come to a focus on the retina, but focus behind it. The muscles of accommodation cannot make the lens sufficiently convex to do its work. An artificial lens is needed, and a properly adjusted convex glass in front of the eye does the work by aiding the lens of the eye.

Authorities—DeWecker and others—place the proportion of hypermetropia existing in internal squint at 75 to 85 out of every 100 cases. On the other hand, in divergent squint (eyes turned outward) there is generally myopia—near-sightedness. Here the antero-posterior diameter of the ball is too long, and concave glasses are needed to prevent the rays converging too far in front of the retina. Divergent squint, however, being so much less common than convergent, I will only speak of the latter variety in this article.

Convergent squint, when caused by hypermetropia, rarely ever manifests itself until the child begins to use its eyes for near objects, at say four to seven years of age. In the effort to read or see close objects it is necessary to increase the convergence of the visual lines to aid the power of accommodation, and the internal rectus muscle participating so frequently in this act, the parents of the child pretty soon notice that it is becoming cross-eyed. They then either stop the child from its studies or do worse, and follow what has been the unfortunate but too common advice of the family physician, "let it alone, as the child will outgrow it," until finally the child will become worse and worse cross-eyed. The worst crossed eye will become useless finally, because we must not lose sight of the fact that they soon learn to suppress the image in the crossed eye, and do not use the eye. And furthermore, even in after years when the squint has been operated upon, they still retain the ungainly habit of ducking the head to one side.

In the large majority of these cases if, in the beginning, the child's eyes had been properly examined and the error of refraction shown up by the ophthalmoscope, and the child been supplied with proper glasses, the eyes would not have become crossed; nor would any operation have been necessary.

Astigmatism, a still more complicated error of refraction, may still further complicate the case. In astigmatism there is irregularity of curvature of some one, or perhaps more than one, meridian of the cornea. This must be corrected by properly applied cylindrical lenses. The scientific management of these cases of errors of refraction calls forth the very highest skill of the oculist, and the successful results obtained are justly regarded as far finer work than the most brilliant handling of the knife. I had the pleasure of spending two months of the past summer attending the clinics of Drs. C. R. Agnew, Pomeroy and Webster at the Manhattan Eye and Ear Infirmary in New York city. I was charmed by the splendid success of these conservative ophthalmologists, especially on this very point. It was a very common sight to see in their clinics young children being relieved of a beginning squint by simply wearing skillfully adjusted glasses.

Of course, if the squint has become established, we must operate; yet it is a very important point to look for the anomaly of refraction, and fit them with glasses also, or the squint may return. The following among some recent cases of young cross-eyed hypermetropes whom I have operated upon will illustrate:

This young lady is aged fourteen and is still at school. She had always had trouble in using her eyes for close work, and both eyes had been crossed since her seventh year. The right eye was very badly crossed. Examination with the ophthalmoscope showed her not only to be very hypermetropic in one eye and slightly myopic in the other, but she was also astigmatic in both eyes, making quite a complicated anomaly of refraction. I operated very freely upon both eyes. By freely I mean that a long conjunctival cut was made, and the sub-conjunctival tissue was freely loosened over the muscle. The operation was instantly successful; but, after two or three weeks, one eye showed signs of again turning inward, when I fitted her with the proper spherical combined with cylindrical glasses, and directed her to wear them constantly. Her vision was thereby improved from $\frac{20}{200}$ and $\frac{20}{50}$ to respectively $\frac{20}{20}$, or normal, and $\frac{20}{30}$. She now pursues her studies easily and with straight eyes.

---

HOW THEY PRACTICE IN INDIA.—An Indian physician was holding forth the other day to some of his brothers of the craft in England. "You sairs in the West," he said, "do not understand the practice of medicine. In my country, if a rajah with nothing of sickness sends for me, I go and I say, 'Sair, your case is a bad one; you will be worse before you are better.' I give him some medicine and I go away. The next day I go again, and I find him heaving like a sea-sick mandarin, and wishing that the son of his mother had never seen the light. 'Sair,' I say, 'I told you so; you have passed a great crisis. There is no more need of medicine. Another sun will see your cure complete.' I then collect my fees, and I go away. When I have cured a few more rajahs I shall come again to your country and take a villa on your little river Thames, with the green turf sloping down to the waterside."—*Ex.*

